# Supplementary material for: Predicting visual function by interpreting a neuronal wiring diagram
Source: Nature. 2024 Oct 2;634(8032):113–23. doi: 10.1038/s41586-024-07953-5 (PMC11446822; doi:10.1038/s41586-024-07953-5)
Supplement: Supplementary file 6 — CRF and ERF predictions for individual TmY4 and TmY9 cells. Analogous to Supplementary Data 3, but for TmY target types. Shown are the top four monosynaptic pathways, the strongest pathway passing through each of the top ten intermediary types (ranking from Extended Data Fig. 7), and the trisynaptic pathway Tm1–TmY–Dm3–TmY (see the section entitled Prediction of spatial normalization). [file 41586_2024_7953_MOESM6_ESM.zip › DataS4/TmY9qΓèÑ/720575940636654328.pdf]

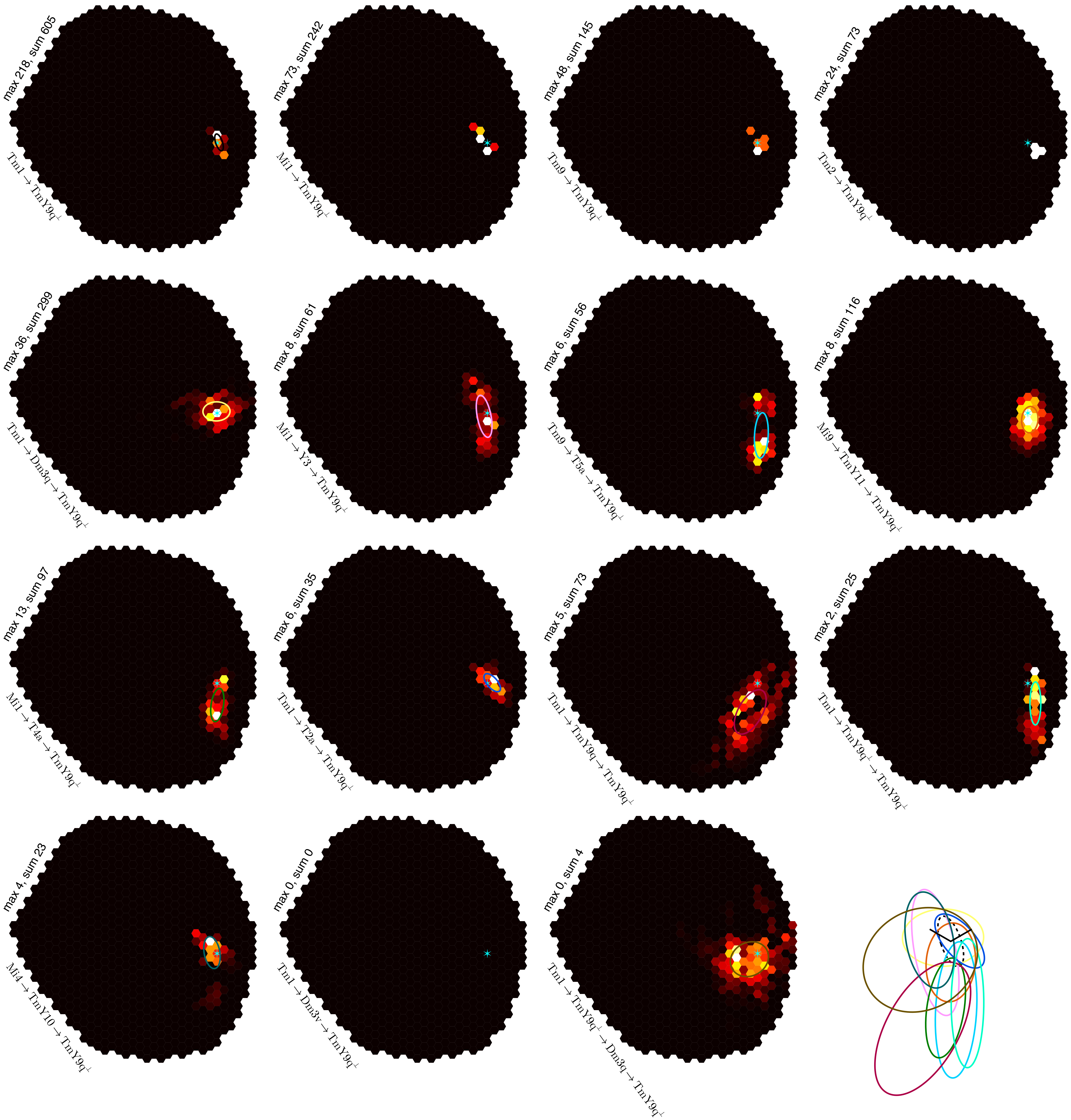

max 218, sum 605
Tm1 → TmY9q⊥
max 73, sum 242
Mi1 → TmY9q⊥
max 48, sum 145
Tm9 → TmY9q⊥
max 24, sum 73
Tm2 → TmY9q⊥
max 36, sum 299
Tm1 → Dm3q → TmY9q⊥
max 8, sum 61
Mi1 → Y3 → TmY9q⊥
max 6, sum 56
Tm9 → T5a → TmY9q⊥
max 8, sum 116
Mi9 → TmY11 → TmY9q⊥
max 13, sum 97
Mi1 → T4a → TmY9q⊥
max 6, sum 35
Tm1 → T2a → TmY9q⊥
max 5, sum 73
Tm1 → TmY9q → TmY9q⊥
max 2, sum 25
Tm1 → TmY9q⊥ → TmY9q⊥
max 4, sum 23
Mi4 → TmY10 → TmY9q⊥
max 0, sum 0
Tm1 → Dm3v → TmY9q⊥
max 0, sum 4
Tm1 → TmY9q⊥ → Dm3q → TmY9q⊥
